# Understanding the interplay between stress, anxiety, and depression and their impact on health in traffic police officers

**DOI:** 10.3389/fpsyt.2025.1580673

**Published:** 2025-06-09

**Authors:** Carlos Ramos-Galarza, Fiamma Flores, Taysha Argoti, Diego D. Díaz-Guerra, Marena de la C. Hernández-Lugo, Yunier Broche-Pérez

**Affiliations:** ^1^ Facultad de Salud y Bienestar, Carrera de Psicología Clínica, Pontificia Universidad Católica del Ecuador, Quito, Ecuador; ^2^ Departamento de Psicología, Universidad Central Marta Abreu de Las Villas, West Palm Beach, Villa Clara, Cuba; ^3^ Department of Behavior Analysis, Prisma Behavioral Center, West Palm Beach, FL, United States

**Keywords:** stress, anxiety, depression, health, traffic police

## Abstract

**Background:**

Traffic police officers are frequently exposed to stress, anxiety, and depression, which adversely impact their health. This study hypothesized that these factors influence the hormonal, muscular, digestive, and cognitive systems of traffic police officers.

**Methods:**

The sample comprised 146 traffic police officers (42 women and 104 men) aged 30 to 38. Gender differences in symptoms were examined, and model fit was assessed using indicators such as chi-square, comparative fit index, root mean square error of approximation (RMSEA), and standardized root means residual (SRMR).

**Results:**

Gender differences were observed, with women reporting more significant issues related to muscular (*t* = 2.77, *p* = .003), hormonal (*t* = 2.29, *p* = .001), and cognitive symptoms (*t* = 1.37, *p* = .08). The models demonstrated a good fit, particularly in the model examining digestive symptoms (CFI.95, RMSEA.06 (.05 -.08), SRMR.04).

**Conclusions:**

The findings indicate a substantial impact of stress, anxiety, and depression on traffic police officers’ health. Psychological support and monitoring are recommended at traffic police stations.

**Practical and academic contribution:**

This research offers essential academic contributions by advancing understanding of the psychophysiological effects of stress, anxiety, and depression in high-stress occupations, using predictive modeling. Practically, the findings support the development of targeted mental health interventions and preventive strategies tailored to traffic police officers, contributing to improved occupational health and job performance.

## 1 Introduction

Stress is an adaptive response of the organism to stimuli perceived as threatening or challenging, which can be physical, emotional, or environmental (1). This response involves physiological, psychological, and behavioral changes to restore homeostasis and facilitate adequate adaptation to external demands (2, 3). However, when stress becomes chronic or excessive, it can significantly impact an individual’s health and well-being, involving complex interactions between various biological and psychological systems. These adverse effects may manifest as symptoms, including cardiovascular and endocrine issues, sleep disorders, and cognitive and emotional difficulties (4–7).

Anxiety, in line with the stress response, is an emotional reaction characterized by feelings of apprehension, unease, and persistent worry (8, 9). While anxiety can be a normal and adaptive response to stressful situations, its intensity and duration distinguish its pathological form and negative impact on an individual’s functionality. This close relationship with stress highlights the importance of differentiating between adaptive and pathological anxiety, especially in contexts where exposure to stressors is frequent and prolonged (10–12).

In the context of police work, anxiety and depression are not only highly prevalent but also critically under-addressed. These conditions often remain undetected due to stigma, lack of mental health infrastructure, and cultural norms that discourage emotional vulnerability. Among traffic police officers, who endure continuous exposure to public stress, aggression, and risk, the development of anxiety and depressive disorders poses a serious threat to occupational performance and personal well-being. Understanding the specific ways these disorders manifest and interact with physiological systems is crucial for early detection and intervention strategies tailored to this workforce. Similarly, depression is a mood disorder marked by persistent sadness, loss of interest in previously enjoyable activities, and a variety of physical and cognitive symptoms (13, 14). Vulnerability to depression may be influenced by genetic, environmental, and neurobiological factors, making this interplay a critical area of mental health research (15–18).

Extensive research has documented the relationship between stress, anxiety, and depression, suggesting that chronic stress can precipitate the onset of depressive and anxious episodes (19–21). Studies indicate that prolonged stress can alter brain chemistry, affecting key neurotransmitters such as adrenaline, serotonin, and dopamine, which are directly linked to mood and motivation (22–24).

Moreover, chronic stress can lead to structural changes in the brain, such as reduced hippocampal volume. This reduction has been associated with deficits in memory and emotional regulation, factors that can significantly contribute to the development of depression and anxiety (25, 26). The connection between stress, anxiety, and depression underscores the need for an integrated approach to address these interrelated conditions. The interdependence of these disorders implies that effective treatment must extend beyond addressing individual symptoms and focus on shared underlying causes (27).

To fully understand how stress contributes to the development of anxiety and depression, it is essential to explore the underlying neurobiological mechanisms. Stress activates a series of physiological responses involving multiple body systems, including the central nervous and endocrine systems (11, 28).

The Hypothalamic-Pituitary-Adrenal (HPA) axis plays a central role in the stress response. In response to a stressor, the hypothalamus releases corticotropin-releasing hormone (CRH), which stimulates the pituitary gland to secrete adrenocorticotropic hormone (ACTH). ACTH then travels through the bloodstream to the adrenal glands, prompting the release of cortisol (29, 30). Cortisol is crucial for regulating metabolism, the immune response, and mood. However, prolonged elevated cortisol levels can lead to neurotoxic effects, particularly in the hippocampus, a brain region involved in memory and emotional regulation (31, 32).

In addition to the HPA axis, other brain structures, such as the amygdala and the prefrontal cortex, are also involved in the stress response (33). The amygdala, essential for emotional assessment and fear responses, can become hyperactive under chronic stress conditions (34, 35). Conversely, the prefrontal cortex, responsible for executive functions and behavioral regulation, may experience reduced volume and functional connectivity, leading to difficulties in decision-making and emotional control (36).

Dysfunction in these systems not only increases vulnerability to anxiety and depression but may also perpetuate a cycle of negative feedback (27). For instance, hyperactivity of the amygdala can heighten threat perception, exacerbating the stress response and perpetuating anxiety (37). At the same time, reduced hippocampal volume can impair the ability to process and overcome negative experiences, contributing to the persistence of depression (38).

Despite a growing body of research on occupational stress among law enforcement officers, few studies have explored the specific psychophysiological manifestations of stress, anxiety, and depression in traffic police—a group uniquely exposed to chronic urban stressors. This study contributes novel insights by focusing on the predictive relationships between psychological factors and distinct symptom domains (muscular, hormonal, cognitive, and digestive), using structural equation modeling. To our knowledge, this is one of the first empirical investigations of these relationships in a Latin American context, particularly within a traffic police population.

### 1.1 Stress, mental health, and cognitive health in police officers

The role of police officers is pivotal in maintaining public safety and order, often placing them in high-stress situations that can significantly impact their mental and physical health (39). The nature of police work inherently involves frequent exposure to potentially traumatic events, high-stakes decision-making, and considerable public scrutiny. These factors contribute to a uniquely stressful occupational environment (40–42).

Emerging research highlights that chronic stress within the police force is associated with a range of adverse health outcomes, including increased risk of cardiovascular diseases, sleep disorders, and mental health conditions such as anxiety, depression, and post-traumatic stress disorder (PTSD) (43). Moreover, the cumulative stress experienced by police officers affects not only their well-being but also their performance and interactions with the community.

The impact of stress on the mental health of police officers is profound and multifaceted. Repeated exposure to traumatic events, such as violent crimes and accidents, can lead to Post-Traumatic Stress Disorder (PTSD) (44), characterized by flashbacks, nightmares, severe anxiety, and uncontrollable thoughts about these events (45, 46). Anxiety and depression are also common among police officers, driven by the constant pressure to perform and the regular encounters with human suffering (47). These mental health conditions can manifest as persistent worry, fear, sadness, and a loss of interest in daily activities. Burnout, a state of emotional, physical, and mental exhaustion, is another significant issue, leading to emotional exhaustion, depersonalization, and a reduced sense of personal accomplishment (48). To cope with their high levels of stress, some officers may turn to substance abuse (49, 50), which can further exacerbate their mental health problems and lead to dependency. The compounded effects of PTSD, anxiety, depression, and burnout can increase the risk of suicidal thoughts and behaviors, with suicide rates among police officers being higher than in the general population (51).

Chronic stress can also impair cognitive functions such as memory, attention, and decision-making, which are crucial during emergency responses (52). Police work often requires accurately recalling details from crime scenes, witness statements, and procedural protocols. Chronic stress can disrupt short-term and long-term memory (53), making it difficult for officers to remember crucial information. This can lead to judgment, reporting, and testimony errors, potentially compromising investigations and legal proceedings.

Attention and concentration are also adversely impacted by stress. Maintaining focus in dynamic and often dangerous environments is critical for police officers. High stress levels can lead to difficulties concentrating, increased distractibility, and a reduced ability to process information quickly and accurately (54). This can hinder an officer’s ability to respond appropriately to unfolding situations, increasing the risk of errors and accidents. Decision-making is another cognitive function that suffers under chronic stress. Police officers are frequently required to make quick decisions under pressure. Stress can impair the prefrontal cortex (55), the brain area responsible for executive functions, including decision-making, problem-solving, and impulse control. When this area is compromised, officers may struggle to assess situations accurately, consider the consequences of their actions, and make sound judgments (56). This can lead to poor decision-making in critical moments, affecting officer and public safety (57).

Furthermore, stress can lead to cognitive fatigue, where mental exhaustion sets in due to prolonged periods of high stress and cognitive load. Cognitive fatigue can manifest as slower reaction times, reduced vigilance, and impaired cognitive flexibility (58), making it harder for officers to adapt to changing circumstances and new information.

### 1.2 Stress and endocrine health in police officers

The impact of stress on the endocrinological health of police officers is profound and multifaceted, with significant implications for their overall well-being. Chronic stress, a shared experience in police work, activates the hypothalamic-pituitary-adrenal (HPA) axis, leading to sustained elevations in cortisol levels (59). This hormone, essential for managing acute stress, becomes detrimental when persistently elevated.

High cortisol levels are associated with various metabolic disorders, including increased appetite, abdominal weight gain, insulin resistance, and type 2 diabetes (60, 61). Furthermore, chronic cortisol elevation contributes to cardiovascular problems such as hypertension and a heightened risk of heart disease and stroke (61). The immunosuppressive effects of cortisol also render police officers more susceptible to infections and slow their recovery processes (62).

In addition to cortisol, the stress response involves the adrenal medulla’s secretion of adrenaline and norepinephrine, hormones that prepare the body for immediate physical action. Chronic activation of this system can lead to persistent cardiovascular strain, exacerbating the risk of long-term heart health issues (46). Stress also disrupts reproductive hormones, leading to menstrual irregularities in female officers and reduced testosterone levels in male officers, affecting fertility and sexual health (63).

Despite the critical importance of this issue, there remains a need for comprehensive studies that explore the specific mechanisms through which occupational stress impacts the health of police officers. Understanding these mechanisms is essential for developing effective interventions and support systems to mitigate the detrimental effects of stress in this population. Although existing literature has documented the high levels of occupational stress among police officers, there is a lack of studies that specifically examine how anxiety and depression interact with physiological symptoms across multiple body systems in traffic police. Furthermore, limited research has applied predictive modeling techniques to analyze these interactions in Latin American populations. This study addresses this gap by using structural equation modeling to evaluate how anxiety, stress, and depression predict cognitive, hormonal, muscular, and digestive symptoms in Ecuadorian traffic police officers.

In this context, the present research has the following objectives: (a) to identify the relationship between depression, anxiety and stress with sociodemographic variables of traffic police officers, (b) to analyze the correlation between depression, anxiety and stress with hormonal, digestive, muscular and cognitive symptomatology present in traffic police officers and (c) to determine the dynamics of explanatory models that consider the interaction of anxiety, stress and depression variables as predictors of hormonal, cognitive, muscular and digestive symptomatology in traffic police officers.

## 2 Method

### 2.1 Participants

The study was conducted with the entire population of traffic police officers in Quito, Ecuador. The sample included 146 officers (42 women and 104 men) aged between 30 and 38 (M = 33.58; SD = 2.11). Regarding marital status, 26.7% were single, 45.9% were married, 6.2% were divorced, 0.7% were separated, 19.2% were in common-law relationships, and 1.3% were widowed.

The inclusion criteria for participants were: (a) being a member of the Metropolitan Transit Agency aged 30 to 45 years and (b) consenting to participate voluntarily by signing an informed consent form approved by the Ethics Committee. The exclusion criteria were: (a) officers assigned to roles other than traffic duties, (b) individuals from vulnerable or disabled groups, (c) female officers who were pregnant, (d) officers outside the specified age range, and (e) officers who chose not to participate voluntarily.

### 2.2 Measuring instruments

The DASS-21 brief scale (64) was used to measure anxiety, stress, and depression. This instrument consists of 21 self-report questions designed to assess behavioral aspects of anxiety, stress, and depression experienced in daily life (65) (**Annex 1**).

Four additional scales were developed specifically for this research to evaluate cognitive, hormonal, digestive, and muscular symptoms. The development process included (a) item generation by the research team, (b) content analysis and cognitive interviews, (c) a preliminary application phase, and (d) the final design and refinement of the scales. Each custom scale was developed using a structured process: (a) literature review and item generation; (b) expert content validation; (c) pilot testing with a subsample (n = 30); and (d) final refinement. Internal consistency was acceptable for all scales (α = .77 to.87). Further details on scale development and psychometric properties are provided in **Annex 2**. A confirmatory factor analysis was conducted for each scale based on its proposed structure, and the results indicated adequate model fit. The fit indices are presented in **Table 1**.

**Table 1:** Confirmatory factor analysis for each scale used in this study.

Model	Chi-square (χ²)	*CFI*	RMSEA (90% CI)	SRMR
Digestive Symptoms	240.77	.95	.06 (0.05–0.08)	0.04
Cognitive Symptoms	237.01	.95	.06 (0.05–0.08)	0.04
Muscular Symptoms	253.54	.94	.07 (0.05–0.08)	0.04
Hormonal Symptoms	271.65	.93	.07 (0.06–0.09)	0.05

The theoretical justification for each item developed in this study to assess the impact of stress on traffic police officers is presented in **Table 2**. The items are classified into four symptom categories—muscular, cognitive, hormonal, and digestive—that are commonly associated with chronic stress. Each justification is grounded in established literature and supports the conceptual validity and practical relevance of the items for evaluating the physiological and psychological effects of occupational stress.

**Table 2:** Theoretical justification of items assessing stress-related symptoms in traffic police officers.

Item	Category	Theoretical Justification
I have felt my body very tense	Muscular Problems	Muscle tension is a common physiological response to chronic stress. In high-stress occupations such as traffic police work, the constant vigilance and physical strain can lead to persistent muscle tightness, which this item aims to capture.
I have experienced headaches	Muscular Problems	Headaches, particularly tension-type headaches, are strongly associated with muscular tension in the neck and shoulders, which are often exacerbated by occupational stress.
I feel back pain	Muscular Problems	Stress-related postural issues and muscle strain from extended periods of standing or movement in traffic duties can manifest as chronic back pain.
I find it difficult to retain information and concentrate	Cognitive Problems	Stress impairs attention and working memory processes, reducing an individual’s ability to focus and retain information, especially under high-pressure environments.
I find it complicated to make decisions	Cognitive Problems	Chronic stress affects the prefrontal cortex, which is essential for executive functions such as decision-making, often leading to indecisiveness or mental fatigue.
I have difficulty understanding	Cognitive Problems	Cognitive overload and fatigue resulting from prolonged stress may lead to impaired comprehension and slower information processing.
I have trouble falling or staying asleep	Hormonal Problems	Stress disrupts the circadian rhythm and increases cortisol levels, contributing to sleep disturbances such as insomnia, which is a common complaint among high-stress professionals.
I have noticed the appearance of acne, pimples, or other skin eruptions	Hormonal Problems	Elevated stress hormones can stimulate sebaceous gland activity, leading to acne and other dermatological issues, which are markers of hormonal imbalance.
I feel that I have lost or gained weight	Hormonal Problems	Stress can disrupt appetite-regulating hormones, leading to changes in eating behaviors and weight fluctuations, often noted in high-stress professions.
I have had digestive problems	Digestive Problems	The gastrointestinal system is highly sensitive to psychological stress, which can cause symptoms such as indigestion, cramping, and irregular bowel movements.
I have felt nauseous or have vomited	Digestive Problems	Stress can activate the autonomic nervous system, triggering nausea and even vomiting, which reflect psychosomatic responses to pressure.
I feel that my stomach loosens frequently	Digestive Problems	Increased stress levels stimulate gut motility and can result in frequent bowel movements or diarrhea, commonly reported in stressful working conditions.

### 2.3 Data analysis plan

The research’s statistical analysis began with descriptive statistics to summarize central tendency and dispersion, characterizing the quantitative data obtained. Following this, internal consistency techniques were employed to assess the reliability of the measurements. Correlational and comparative analyses were then conducted to explore the associations between sociodemographic and research variables. Finally, structural equation modeling was applied to evaluate the goodness of fit of the proposed models. Statistical analyses were carried out using SPSS and AMOS software.

### 2.4 Research setting and procedure

This research was conducted in Ecuador, a Latin American country with a population of over 16 million people. Ecuador operates under a capitalist economic system and uses the United States dollar as its official currency. Most of the population is Catholic, and its educational system resembles those found in other countries in the region (66). Given these social, academic, and economic characteristics, the findings of this study may offer insights into the stress, anxiety, and depression experienced by traffic police officers in similar contexts.

The research began with the design phase and received approval from the Ethics Committee for Research on Human Beings at Pontificia Universidad Católica del Ecuador (Code: EO-18-2023, V2). Following this, the research team visited the traffic police station to explain the study’s objectives, ensure voluntary participation, and obtain informed consent from the officers. The instruments were then administered in classrooms provided by the traffic police station. All data collection was conducted anonymously in a distraction-free environment, ensuring the physical and psychological well-being of the participants. The data collection took place between October 2023 and January 2024. After completing data collection, the database was created, statistical analyses were performed, and the final report was drafted.

## 3 Results

### 3.1 First research objective: relationship between depression, anxiety, and stress with sociodemographic variables

The first step in the statistical analysis was to identify the values of central tendency and dispersion. Subsequently, the internal consistency value of each scale was analyzed, which contributed to understanding the reliability of the measurements made (see **Table 3**).

**Table 3:** Descriptive values of research variables.

Variables	N	Min	Max	M (SD)	Reliability of the scale
Depression	146	0	20	5.53 (5.15)	*α* = .90
Anxiety	146	0	20	5.68 (4.62)	*α* = .87
Stress	146	0	21	8.07 (4.89)	*α* = .88
Muscular symptoms	146	0	9	3.86 (2.36)	*α* = .77
Cognitive symptoms	146	0	9	2.50 (2.33)	*α* = .87
Hormonal symptoms	146	0	9	2.99 (2.29)	*α* = .73
Digestive symptoms	146	0	9	2.56 (2.44)	*α* = .83

M, mean; SD, Standard deviation.

The following statistical analysis sought to identify whether there are differences between men and women on measures of depression, anxiety, stress, muscle symptoms, cognitive symptoms, hormonal symptoms, and digestive symptoms. The results showed equality in the affectation of the variables except for hormonal, cognitive, and muscular symptoms, which generate more significant problems in women (see **Table 4**).

**Table 4:** Comparisons between men and women.

Variable	Gender	M (SD)	SD	t-test	Effect Size
Depression	Female	6,29 (5,72)	,884	*t* (144) = 1.12, *p* = .13	*d* = .19
	Male	5,23 (4,89)	,480		
Anxiety	Female	6,24 (4,62)	,713	*t* (144) = .17, *p* = .35	*d* = .03
	Male	5,45 (4,63)	,454		
Stress	Female	8,81 (4,71)	,728	*t* (144) = 1.16, *p* = .12	*d* = .19
	Male	7,77 (4,95)	,486		
Muscular Symptoms	Female	4,69 (2,27)	,352	*t* (144) = 2.77, *p* = .003	*d* = .46
	Male	3,52 (2,31)	,227		
Cognitive Symptoms	Female	3,10 (2,72)	,420	*t* (144) = 1.97, *p* = .02	*d* = .33
	Male	2,26 (2,13)	,209		
Hormonal Symptoms	Female	3,67 (2,34)	,362	*t* (144) = 2.29, *p* = .01	*d* = .38
	Male	2,72 (2,22)	,218		
Digestive Symptoms	Female	3,00 (2,37)	*,367*	*t (144) = 1.37, p = .08*	*d* = .23
	Male	2,38 (2,46)	*,242*		

The next analysis examined the relationship between the seven variables assessed in the investigation and the participants’ age or marital status. No statistically significant associations were found between the variables mentioned (*r* = .01 -.09; *p* = .28 -.86).

### 3.2 Second research objective: relationship between depression, anxiety, and depression with digestive, hormonal, cognitive, and muscular symptomatology

The fourth statistical analysis sought to identify the correlation between the research variables. The results indicate relationship values in a magnitude between medium and large. **Table 5** shows these results.

**Table 5:** Correlation between variables.

Variables	1	2	3	4	5	6	7
1. Depression	-						
2. Anxiety	.817^**^	-					
3. Stress	.802^**^	.801^**^	-				
4. Muscular Symptoms	.655^**^	.662^**^	.754^**^	-			
5. Cognitive Symptoms	.747^**^	.720^**^	.674^**^	.609^**^	-		
6. Hormonal Symptoms	.629^**^	.693^**^	.672^**^	.687^**^	.644^**^	-	
7. Digestive Symptoms	.640^**^	.704^**^	.671^**^	.705^**^	.566^**^	.737^**^	-

^**^(*p*<.001).

### 3.3 Third research objective: analysis of the hypothesized explanatory models

**Figure 1:**
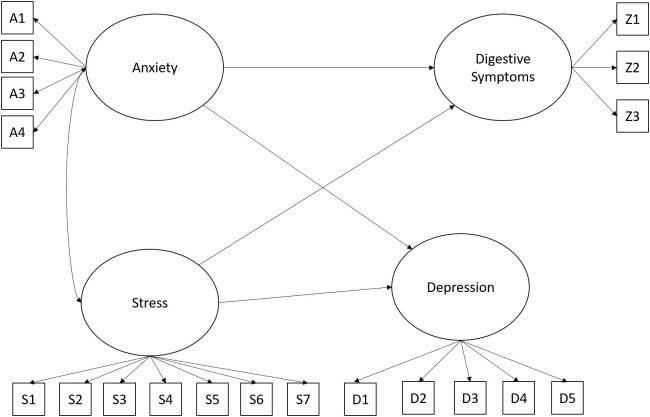
Hypothesized model for understanding digestive symptoms. In all figures, the observed variables representing each latent construct correspond to the individual items that make up the respective factor. The complete list of these items is provided in **Annexes 1** and **2**.

The first model proposed anxiety, stress, and depression as predictor variables of the digestive symptoms of the police officers participating in the research. Adequate fit values were found for the hypothesized model *x^2^*_(146)_ = 240.77, *p* = .001; *CFI*.95; *RMSEA*.06 (.05 -.08); *SRMR*.04. **Figure 1** shows the hypothesized model.

**Figure 2:**
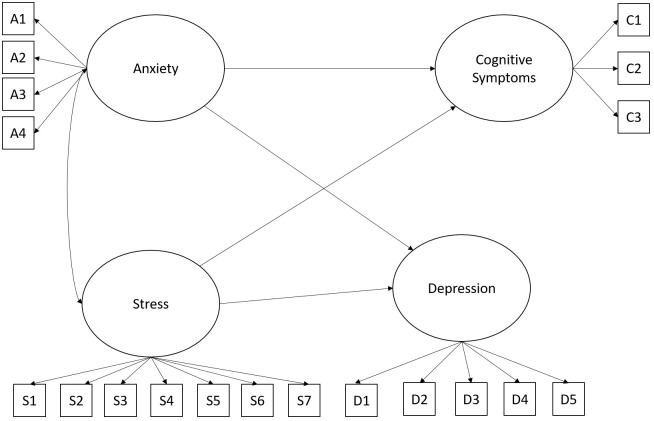
Hypothesized model for understanding cognitive symptoms. In all figures, the observed variables representing each latent construct correspond to the individual items that make up the respective factor. The complete list of these items is provided in **Annexes 1** and **2**.

In the following statistical analysis, we tested the goodness of fit of the second hypothesized model, in which the variables anxiety, stress, and depression are related to explain the cognitive symptoms of the evaluated police officers. The values found suggest an adequate model fit *x^2^* (146) = 237.01, *p* = .001; *CFI*.95; *RMSEA*.06 (.50 -.80) and *SRMR*.04. **Figure 2** shows the hypothesized model.

**Figure 3:**
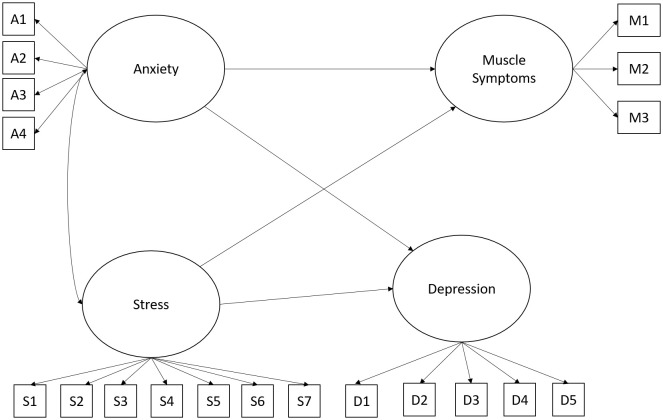
Hypothesized model for understanding muscle symptoms. In all figures, the observed variables representing each latent construct correspond to the individual items that make up the respective factor. The complete list of these items is provided in **Annexes 1** and **2**.

In the third proposed model, the interaction of anxiety, stress, and depression was considered a causal factor in muscle symptoms. In the statistical analysis performed, adequate levels of fit were found for this model: x2 (146) = 253.54, p = .001; CFI.94; RMSEA.07 (.05 -.08); SRMR.04. **Figure 3** shows the proposed model.

**Figure 4:**
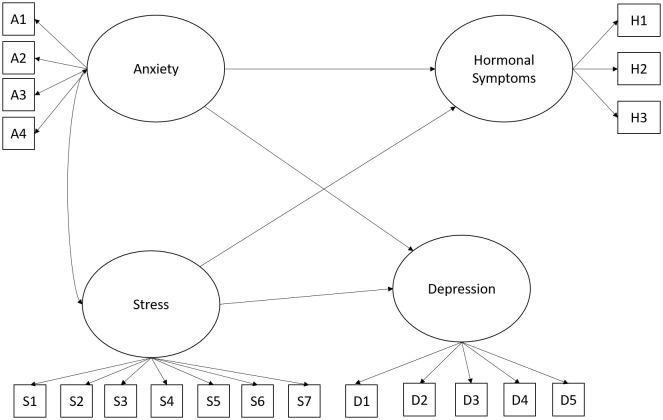
Hypothesized model for understanding hormonal symptoms. In all figures, the observed variables representing each latent construct correspond to the individual items that make up the respective factor. The complete list of these items is provided in **Annexes 1** and **2**.

The fourth hypothesized model proposed that the variables anxiety, stress, and depression are factors that would explain the presence of hormonal symptoms in the police officers participating in the research. Adequate levels of fit were found for the proposed model x2 (146) = 271.65, p = .001; CFI.93; RMSEA.07 (.06 -.09) and SRMR.05. **Figure 4** shows the proposed model.

## 4 Discussion

This study explored the psychophysiological responses to stress among police officers in Quito, Ecuador, revealing significant insights into the impact of stress, anxiety, and depression on both mental and physical health. Our detailed analyses of psychological and physiological variables provided a comprehensive understanding of the health challenges faced by this occupational group.

The statistical analyses demonstrated adequate internal consistency for the scales used, reinforcing the validity of our measurements. Correlation analyses revealed moderate to strong relationships among the variables studied, aligning with previous research that links anxiety, stress, and depression with various psychological and physical symptoms (67–69).

Our findings indicate notable gender differences: female officers reported more severe muscular, cognitive, and hormonal symptoms compared to their male counterparts. This suggests that female officers may be exposed to distinct occupational stressors affecting their health (70–72). However, no significant differences in anxiety, stress, or depression were observed across other sociodemographic variables, such as age or marital status, highlighting common challenges within the police environment. This observation is consistent with prior research indicating that female officers face unique stressors, including discrimination and harassment, which exacerbate stress and adverse symptoms (73, 74).

Our study’s findings corroborate earlier research that connects stress with physical and psychological symptoms in high-pressure occupations. Early identification of these symptoms could enhance individual well-being and job performance among police officers (39, 75).

The observed gender differences, with female officers reporting greater muscular, hormonal, and cognitive symptoms, may be influenced by multiple mechanisms. Biological factors such as hormonal fluctuations, alongside psychosocial stressors—including workplace discrimination, role strain, and insufficient institutional support—could exacerbate vulnerability among female officers. Prior studies have documented these disparities, emphasizing the need for gender-sensitive interventions within police institutions (70, 72, 74).

The structural equation models proposed in this study identified anxiety, stress, and depression as significant predictors of digestive, cognitive, muscular, and hormonal symptoms. The robustness of these models underscores the need for holistic approaches to occupational health that address mental and physical aspects (68, 71, 76).

The first model showed that anxiety, stress, and depression significantly predict digestive symptoms, with fit indices indicating a good model fit. This finding is consistent with literature linking stress and anxiety to gastrointestinal issues (76). Regarding cognitive symptoms, the second model identified anxiety, stress, and depression as significant predictors of cognitive symptoms, such as concentration and memory problems. The model demonstrated a good fit, aligning with studies showing that stress affects cognitive function (47).

The superior model fit observed for digestive symptoms may be attributable to the gut-brain axis, a bi-directional communication system linking emotional and cognitive centers of the brain with peripheral intestinal functions. Psychological stress and anxiety can alter gastrointestinal function through neuroendocrine signaling and microbiome dysregulation, contributing to symptoms like abdominal discomfort, nausea, and altered bowel habits (29). This hypothesis supports the heightened sensitivity of digestive systems to psychological distress.

The third model found that anxiety, stress, and depression are significant predictors of muscular symptoms, including muscle pain and stiffness. The model fit was adequate and consistent with research linking stress to muscular health (77).

The fourth model indicated that anxiety, stress, and depression predict hormonal symptoms, which is also a good model fit. This finding aligns with the literature on the impact of stress and anxiety on hormonal health (73). The close connections identified between psychological stressors and various physical and psychological symptoms emphasize the need for integrated approaches to address these issues. Preventive and therapeutic strategies should be tailored to the specific needs of police officers, incorporating mental and physical health considerations as integral components of occupational well-being (77, 78).

Addressing anxiety, stress, and depression should be central to improving the health and performance of police officers. Implementing stress management programs, such as mindfulness and physical exercise, and fostering supportive work environments can significantly enhance officers’ well-being (79, 80). The interconnectedness of mental and physical health highlights the necessity of a comprehensive approach to occupational health in law enforcement.

### 4.1 Limitations and future research

This study’s findings are based on data collected from a specific Latin American city, which may limit the generalizability of the results. However, Quito’s characteristics as a globalized city with significant traffic congestion suggest that these findings could be relevant to other large, chaotic urban environments.

While this study was conducted in Quito, Ecuador—a large, urban, and high-traffic environment—the results may not be fully generalizable to rural contexts or regions with different socio-cultural frameworks and policing structures. Differences in institutional policies, economic development, and cultural attitudes toward mental health may influence the manifestation and reporting of stress-related symptoms. Comparative studies involving police officers from diverse regions, including the Global North and rural Latin American contexts, are recommended to explore these variables further.

Due to the cross-sectional design of the study, causal relationships between stress, anxiety, depression, and physiological symptoms cannot be inferred. The simultaneous measurement of predictors and outcomes poses limitations on temporal precedence. Longitudinal and experimental designs are encouraged in future research to establish causality and monitor symptom progression over time.

Future studies should focus on developing and evaluating interventions aimed at reducing stress, anxiety, and depression among traffic police officers. Additionally, psychoeducational programs could be implemented to increase officers’ awareness of how negative psychological factors impact their health and provide strategies to mitigate these effects.

## Data Availability

The datasets presented in this study can be found in online repositories. The names of the repository/repositories and accession number(s) can be found below: https://doi.org/10.17632/58yntjyhh2.1.
