# Supplementary material for: Understanding the interplay between stress, anxiety, and depression and their impact on health in traffic police officers
Source: Front Psychiatry. 2025 Jun 9;16:1580673. doi: 10.3389/fpsyt.2025.1580673 (PMC12183194; doi:10.3389/fpsyt.2025.1580673)
Supplement: Supplementary file 1 [file DataSheet1.docx]

**Annex 1.** Measurement scale employed to asses stress, anxiety, and depression.

| Item Number | Category | Abbreviation used in figures | Item | Item (Spanish) |
| --- | --- | --- | --- | --- |
| 1 | Stress | S1 | I found it hard to wind down | Me ha costado mucho descargar la tensión |
| 2 | Anxiety | A1 | I was aware of dryness of my mouth | Me di cuenta de que tenía la boca seca |
| 3 | Depression | D1 | I couldn´t experience any positive feeling at all | No podía sentir ningún sentimiento positivo |
| 4 | Anxiety | A2 | I experienced breathing difficulty | Se me hizo difícil respirar |
| 5 | Depression | D2 | I found it difficult to work up the initiative to do things | Se me hizo difí­cil tomar la iniciativa para hacer cosas |
| 6 | Stress | S2 | I tended to over-react to situations | Reaccioné exageradamente en ciertas situaciones |
| 7 | Anxiety | A3 | I experienced trembling (e.g. in the hands) | Sentí que mis manos temblaban |
| 8 | Stress | S3 | I felt that I was using a lot of nervous energy | He sentido que estaba gastando una gran cantidad de energí­a |
| 9 | Anxiety | A4 | I was worried about situations in which I might panic and make a fool of myself | Estaba preocupado por situaciones en las cuales podía tener pánico o en las que podrí­a hacer el ridí­culo |
| 10 | Depression | D3 | I felt that nothing could cheer me up | He sentido que no habí­a nada que me ilusionara |
| 11 | Stress | S4 | I felt I was close to panic | Me he sentido inquieto |
| 12 | Stress | S5 | I found it difficult to relax | Se me hizo difí­cil relajarme |
| 13 | Depression | D4 | I felt down-hearted and blue | Me sentí triste y deprimido |
| 14 | Stress | S6 | I was intolerant of anything that kept me from getting on with what I was doing | No toleré nada que no me permitiera continuar con lo que estaba haciendo |
| 15 | Depression | D5 | I couldn´t seem to get any enjoyment out of the things I did | No me pude entusiasmar por nada |
| 16 | Stress | S7 | I found myself getting agitated | He tendido a sentirme enfadado con facilidad |
