# Supplementary material for: Understanding the interplay between stress, anxiety, and depression and their impact on health in traffic police officers
Source: Front Psychiatry. 2025 Jun 9;16:1580673. doi: 10.3389/fpsyt.2025.1580673 (PMC12183194; doi:10.3389/fpsyt.2025.1580673)
Supplement: Supplementary file 2 [file DataSheet2.docx]

**Annex 2.** Measurement Scales Employed to asses muscular, cognitive, digestive and hormonal problems.

| Item Number | Category | Abbreviation used in figures | Item | Item (Spanish) |
| --- | --- | --- | --- | --- |
| 1 | Muscular Problems | M1 | I have felt my body very tense | He sentido mi cuerpo muy tenso |
| 2 | Muscular Problems | M2 | I have experienced headaches | He sentido dolores de cabeza |
| 3 | Muscular Problems | M3 | I feel back pain | Siento dolor de espalda |
| 4 | Cognitive Problems | C1 | I find it difficult to retain information and concentrate | Se me dificulta retener información y concentrarme |
| 5 | Cognitive Problems | C2 | I find it complicated to make decisions | Se me hace complicado tomar decisiones |
| 6 | Cognitive Problems | C3 | I have difficulty understanding | Tengo dificultad para comprender |
| 7 | Hormonal Problems | H1 | I have trouble falling or staying asleep | Se me dificulta conciliar o mantener el sueño |
| 8 | Hormonal Problems | H2 | I have noticed the appearance of acne, pimples, or other skin eruptions | He notado el aparecimiento de acné, espinillas u otras erupciones cutáneas |
| 9 | Hormonal Problems | H3 | I feel that I have lost or gained weight | Siento que he perdido o ganado peso |
| 10 | Digestive Problems | Z1 | I have had digestive problems | He tenido problemas digestivos |
| 11 | Digestive Problems | Z2 | I have felt nauseous or have vomited | He tenido sensación de náusea o vómito |
| 12 | Digestive Problems | Z3 | I feel that my stomach loosens frequently | Siento que se me afloja el estómago con frecuencia |
